# Serodiagnosis of Anti-glomerular Basement Membrane Disease Using a Newly Developed Chemiluminescence Immunoassay

**DOI:** 10.3389/fmed.2022.915754

**Published:** 2022-07-04

**Authors:** Alexander Kühnl, Lea Hartwig, Cornelia Dähnrich, Wolfgang Schlumberger

**Affiliations:** Institute for Experimental Immunology, EUROIMMUN Medizinische Labordiagnostika AG, Lübeck, Germany

**Keywords:** anti-GBM, glomerulonephritides, chemiluminescence immunoassay, CLIA, ChLIA, renal autoimmune diseases

## Abstract

Circulating autoantibodies directed against the kidney glomerular basement membrane (GBM) antigens are important markers in the diagnosis and monitoring of autoimmune glomerulonephritides, including the classic Goodpasture's syndrome. Rapid and reliable diagnostic tools for the detection of anti-GBM autoantibodies are crucial as anti-GBM disease can progress rapidly and, if too late or incorrectly diagnosed, can have serious, even fatal consequences. The performance of the newly developed standardized chemiluminescence immunoassay (ChLIA) was evaluated in comparison with the established Anti-GBM ELISA (IgG) (EUROIMMUN). For the assessment of its diagnostic performance, sera from 67 clinically characterized anti-GBM disease patients and 221 disease controls were analyzed. The clinical sensitivity of the Anti-GBM ChLIA (IgG) reached 100% at a specificity of 98.6%. The Anti-GBM ELISA (IgG) performance was less sensitive (89.6%) without any positive findings in the control group, indicating a specificity of 100%. Both methods were homogeneous (κ = 0.901). The Anti-GBM ChLIA (IgG) represents a promising alternative tool for accurate anti-GBM assessment in routine diagnostic settings with the advantage of rapid turnaround time and fully automated random-access processing.

## Introduction

In autoimmune glomerulonephritis, autoantibodies are directed against antigens expressed in the glomerular basement membrane (GBM) of the kidney glomeruli, causing an inflammation eventually leading to glomerulosclerosis and dialysis-dependent kidney failure (1–4). Progressive forms of such anti-GBM disease may cause life-threatening circumstances, especially in combination with lung injury (5). The primary target antigen is the non-collagenous (NC1) region of the alpha-3 chain of the network-structured type IV collagen in the basement membrane lamina densa (6, 7). In routine diagnostics, this antigen is thus utilized as substrate by various immunoassay formats, most notably indirect immunofluorescence tests and ELISA, for the detection of anti-GBM autoantibodies to support diagnosis of anti-GBM disease. A recent literature-based meta-analysis confirmed the potential high sensitivity and specificity in the diagnosis of anti-GBM disease (8).

Anti-GBM antibodies, predominantly of class IgG, are found in serum in 90% of patients with anti-GBM glomerulonephritides (3). Such antibodies also exist in healthy individuals, but the immune system prevents substantial formation and circulation most importantly by regulatory T cells (4, 9, 10). Clinical progression of the disease correlates with antibody concentration (11, 12) and its use in patient monitoring increases the demand for high-quality immunoassays with improved performance in terms of sensitivity, measuring range and standardized quantification. In this respect, caveats were reported for ELISA (13). Hence, rapid and reliable diagnostic tools are crucial. Besides diagnosis, there is also the need for more sufficient treatments (4).

Chemiluminescence immunoassays (ChLIA) combined with bead technology and random-access automation open new opportunities for fast, sensitive, and accurate autoantibody detection (14, 15). Here, we evaluated the diagnostic performance of a newly developed Anti-GBM ChLIA (IgG) for detection of anti-GBM autoantibodies.

## Methods

### Patients and Samples

The study included 67 serum samples from anti-GBM disease patients collected from multiple institutions as summarized in Table 1. A total of 221 disease control sera were collected from patients with other relevant systemic autoimmune disorders, including granulomatosis with polyangiitis, microscopic polyangiitis, systemic lupus erythematosus, IgA nephropathy, rheumatoid arthritis, Sjögren's syndrome, ulcerative colitis, and Crohn's disease. These samples were obtained from a variety of clinical sites, as summarized in Table 1. Patients were diagnosed according to local applied guidelines in accordance with internationally accepted criteria. Anti-GBM disease diagnosis was based on confirmation by biopsy staining and/or on serological anti-GBM detection in conjunction disease manifestations according to Hellmark and Segelmark (1).

**Table 1:** Patient characteristics.

**Panel**	**Origin**	**Quantity**	**Male/female**	**Mean age (range)**
Anti-GBM disease		67		
	III. Department of Medicine, University Medical Center Hamburg-Eppendorf, Hamburg, Germany^a^	9	6/3	51 (18–76)
	Department of Rheumatology and Immunology, Hannover Medical School, Hannover, Germany^a^	1	0/1	68
	Department of Pathology, Massachusetts General Hospital, Boston, Massachusetts, USA^a^	16	6/10	58 (19–81)
	Renal Division, Department of Medicine, Peking University First Hospital, Beijing, China^b^	38	15/10^c^	49 (20–78)
	Center for Diagnostics, Department of Clinical Immunology and Transfusion Medicine, Region of Östergötland, Faculty of Medicine and Health Sciences, Linköping University, Linköping, Schweden^b^	3	3/0	77 (74–82)
Disease controls		221		
Granulomatosis with polyangiitis	III. Department of Medicine, University Medical Center Hamburg-Eppendorf, Hamburg, Germany	30	25/5	61 (28–80)
Microscopic polyangiitis	III. Department of Medicine, University Medical Center Hamburg-Eppendorf, Hamburg, Germany	30	22/8	63 (41–80)
Systemic lupus erythematosus	III. Department of Medicine, University Medical Center Hamburg-Eppendorf, Hamburg, Germany	15	4/11	39 (22–69)
Systemic lupus erythematosus	Department of Rheumatology and Immunology, Hannover Medical School, Hannover, Germany	20	5/15	42 (19–78)
IgA nephropathy	III. Department of Medicine, University Medical Center Hamburg-Eppendorf, Hamburg, Germany	26	20/6	49 (19–80)
Rheumatoid arthritis	Department of Rheumatology and Immunology, Hannover Medical School, Hannover, Germany	20	7/13	61 (33–86)
Sjögren's syndrome	Department of Rheumatology and Immunology, Hannover Medical School, Hannover, Germany	20	1/19	53 (16–77)
Ulcerative colitis	Molecular Gastroenterology, University Hospital Schleswig-Holstein, Lübeck, Germany	30	12/18	40 (17–73)
Crohn's disease	Molecular Gastroenterology, University Hospital Schleswig-Holstein, Lübeck, Germany	30	5/25	43 (20–77)

^a^*Diagnosis confirmed by biopsy*.

*^b^Diagnosis according to Hellmark and Segelmark (1): “detection of anti-GBM in tissues or circulation in conjunction with alveolar or glomerular disease”*.

*^c^Gender of 13 patients was unknown*.

Sera were stored at −20°C. Serological analyses were performed blinded to clinical data. Individual and ethical approval was not mandatory for this retrospective study as patient data and (leftover) samples were used anonymously.

### Immunoassays for the Detection of Anti-GBM Autoantibodies

The Anti-GBM ChLIA (IgG) (EUROIMMUN Medizinische Labordiagnostika AG, Lübeck, Germany) is based on magnetic beads coated with recombinant GBM antigen, which is composed of the NC1 region type IV collagen. The assay was performed on a fully automated random-access analyzer (EUROIMMUN) as previously described (14). Measurement output is chemiluminescent units per milliliter (CU/ml) and results ≥10 CU/ml were considered as positive. The cut-off value was determined as the mean of healthy controls +5 standard deviations based on 200 samples from apparently healthy individuals.

The Anti-GBM ELISA (IgG) (EUROIMMUN) was performed according to the manufacturer's instruction. Antibody concentrations in relative units per milliliter (RU/ml) were obtained using a standard curve based on three calibrators provided in the kit. As recommended by the manufacturer's protocol, the cut-off for positivity was defined as ≥20 RU/ml.

### Statistics

The data were evaluated statistically using GraphPad Prism 6, GraphPad Prism QuickCalcs (GraphPad Software Inc., La Jolla, CA, USA) and SigmaPlot 13.0 (SSI, San Jose, CA, USA). Sensitivity was calculated as the proportion of anti-GBM disease samples identified as positive by the respective assay. Specificity was calculated as the proportion of negative results among disease control samples. To examine the discriminatory ability of the assays, receiver-operating characteristics (ROC) curve analysis was carried out. Cohen's kappa test was performed to analyze the agreement between portions, with kappa (κ) values corresponding to almost perfect (0.81–1.00), substantial (0.61–0.80), moderate (0.41–0.60), fair (0.21–0.40), slight (0.01–0.20), and no (≤ 0) agreement. Spearman's rank correlation test was used to determine the degree of correlation between assays. *P*-values < 0.05 were considered significant.

## Results

### Diagnostic Performance Characteristics of Anti-GBM ChLIA (IgG)

Clinical sensitivity and specificity were assessed in 67 anti-GBM disease patients and 221 disease controls, respectively. In these cohorts, the ChLIA had a sensitivity of 100% and specificity of 98.6% (Table 2). Three control samples were found positive and originated from patients diagnosed with granulomatosis with polyangiitis, microscopic polyangiitis, and IgA nephropathy, respectively.

**Table 2:** Comparison of the anti-GBM diagnostic assay performance: ELISA (EUROIMMUN) vs. ChLIA (EUROIMMUN).

**Panel**	***N***	**Anti-GBM IgG positive**	
		**ELISA**	**ChLIA**
Area under the curve (95% CI)		0.999 (0.997–1.000)	1.000 (1.000–1.000)
Anti-GBM disease	67	60	67
Sensitivity (95% CI)		89.6% (79.7–95.7)	100% (94.6–100.0)
Disease controls	221	0	3
Specificity (95% CI)		100% (98.3–100.0)	98.6% (96.1–99.7)

Assessment of analytical assay characteristics included precision testing. For positive samples, coefficients of variation (CV) were calculated as 1.2–3.3% (intra-lot) and 1.6–4.2% (inter-lot). Furthermore, no interference was observed for hemolyzed, lipaemic or icteric samples with up to 10 mg/ml hemoglobin, 20 mg/ml triglycerides and 0.4 mg/ml bilirubin, respectively. The Anti-GBM ChLIA demonstrated linearity within a measurement range of 3.8–517.3 CU/ml.

### Comparison Between ChLIA and ELISA

**Figure 1:**
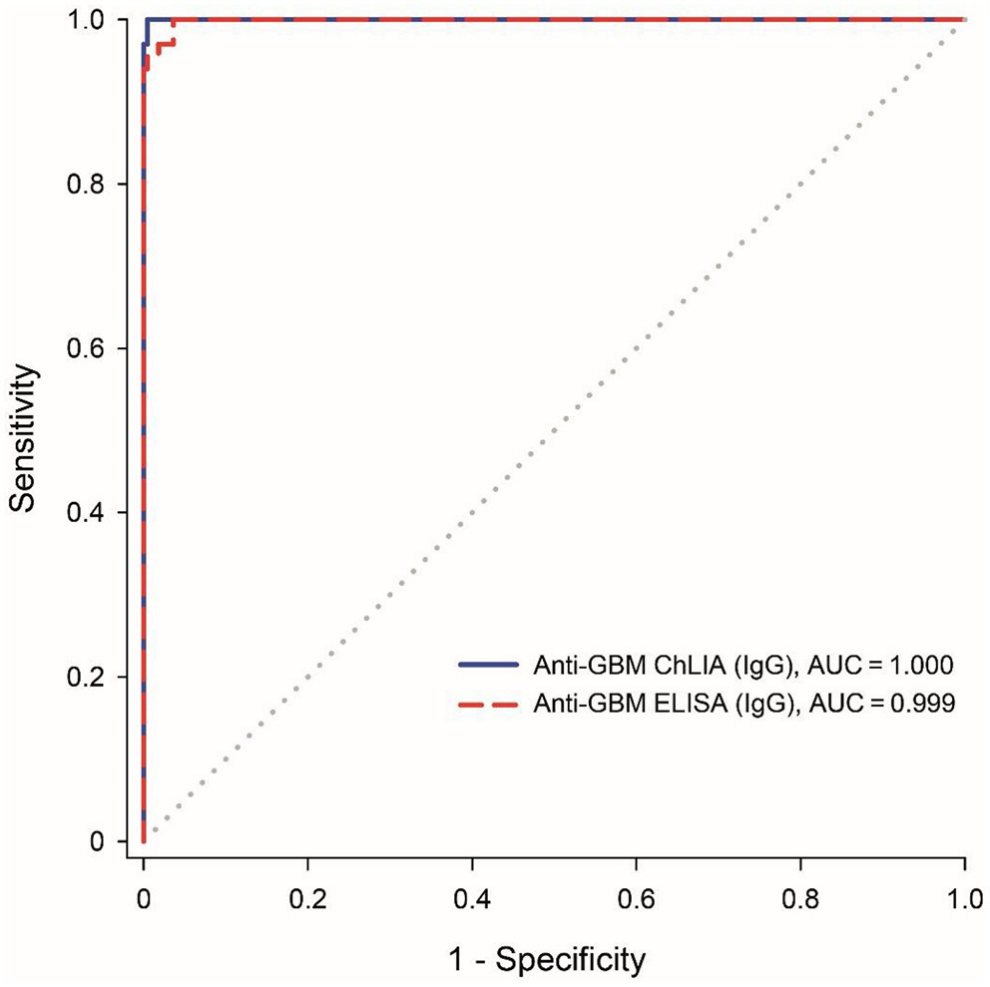
Assay comparison using receiver operating characteristics (ROC) curve analysis for the discrimination between anti-GBM patients (*n* = 67) and disease controls (*n* = 221). The diagonal line indicates no discrimination (area under the curve: 0.5).

In a comparison to the Anti-GBM ELISA (IgG) summarized in Table 2, the Anti-GBM ChLIA (IgG) showed a higher detection rate. While 67 out of 67 anti-GBM disease related samples were determined to be positive with Anti-GBM ChLIA (IgG), 60 out of 67 resulted positive in ELISA (sensitivity 89.6%). None of the control samples had a positive outcome with ELISA (specificity 100%). Thus, the qualitative results of the two assays had a positive agreement of 100% (95% CI: 94.0–100.0%) and a negative agreement of 95.6% (95% CI: 92.1–97.9%). ROC curve analysis revealed high areas under the curve for ChLIA (1.000) as well as ELISA (0.999) (Figure 1; Table 2).

**Figure 2:**
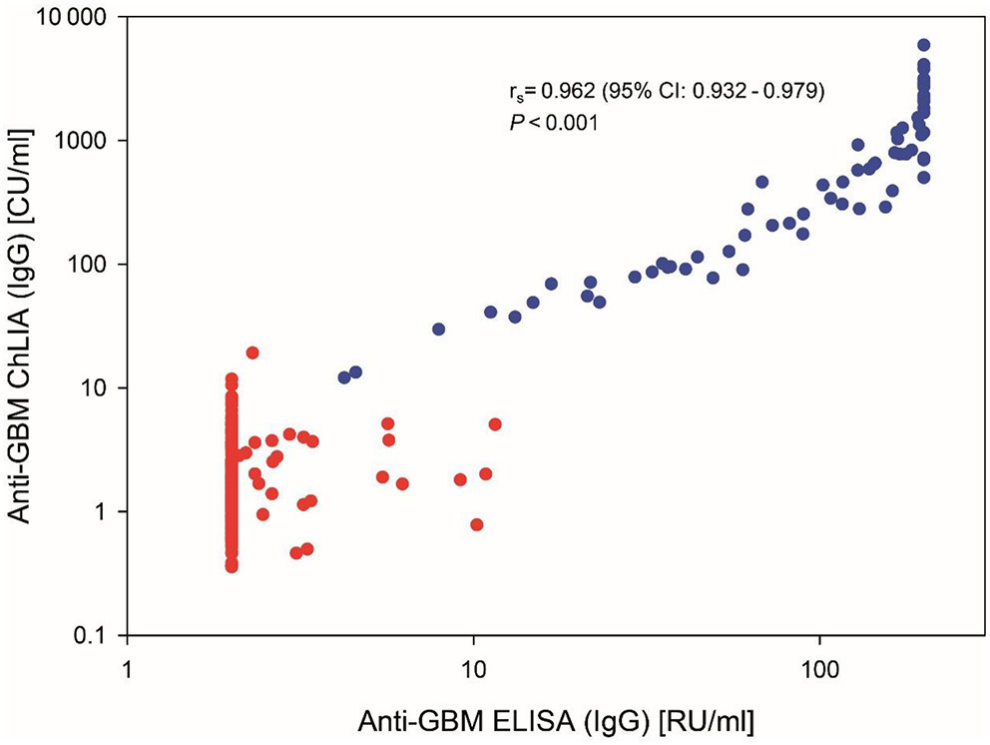
Correlation between anti-GBM levels in 288 serum samples measured by ChLIA vs. ELISA. Axes are displayed in logarithmic scale. Anti-GBM disease samples are depicted with blue data points, control samples in red. Correlation coefficient and *P*-value were calculated using the Spearman's rank correlation.

The almost perfect agreement was also reflected by a κ-value of 0.901 (95% CI: 0.841–0.961) and by a strong significant correlation between the assays' quantitative results (*r*_s_ = 0.962, 95% CI: 0.932–0.979, *P* < 0.001; Figure 2).

## Discussion

The obtained analytical validation data indicate a high-quality of the novel Anti-GBM ChLIA (IgG). An increased detection rate compared to the Anti-GBM ELISA (IgG) was found for the clinically relevant performance testing. In turn, the ELISA yielded a slightly (1.4%) higher specificity in the applied disease control group of clinically characterized patient samples. However, this could likewise be related to the lower detection rate compared to the ChLIA. Two out of three patient samples from the disease control group that resulted anti-GBM positive by ChLIA were diagnosed with anti-neutrophil cytoplasm antibodies (ANCA) associated vasculitis. Both were negative with ELISA as well as indirect immunofluorescence test. It is well-accepted that double positivity of ANCA and anti-GBM occurs in a subset of patients (16, 17). Anticipating the possibility of double positivity, the potential need of these patient for aggressive early treatment as well as careful follow-up due to the higher likelihood of relapses should be considered (17). The result of the ChLIA should not necessarily be considered as false positive, but solely based on the serological data, without proven clinical evidence, it cannot be ruled out either. The same applies to the anti-GBM ChLIA finding in a sample from an IgA nephropathy patient. Some rare reports on the concurrence of anti-GBM nephritis and IgA nephropathy are available (18). Due to the retrospective character of this study, no further investigations about clinical and serological manifestations in these patients are feasible. Future studies could provide further insights and moreover address the need for comparisons among different immunoassays (8). In addition, limitations of the present study could be addressed, for example with regard to sample selection. The diagnostic strategies of the clinical sites that contributed to the anti-GBM patient samples were heterogeneous, using biopsy staining and/or serological confirmation. Restriction to the latter, for example, would exclude discrepant cases where anti-GBM is only detectable *via* indirect immunofluorescence staining. Since it cannot be excluded that our sample collection was skewed toward seropositive anti-GBM disease patients, sensitivity might be overestimated. Future assessment of routine samples in a uniform and consecutive manner, including rare seronegative cases, would avoid any selection bias, thus increasing the value of performance evaluation. Furthermore, the inclusion of follow-up samples could address disease monitoring besides diagnosis.

Since the principal antigen in ChLIA and ELISA is the same, the higher detection rate in ChLIA could be due to the carrier on which this antigen is presented. Possibly, bead-coupled antigen used in ChLIA enables a higher accessibility to autoantibodies in the surrounding fluid phase compared to ELISA microtiter plates. Moreover, ChLIA has the advantage of higher dynamic measurement range. However, antigen preparations differ between ELISA and ChLIA, which could also contribute to the higher detection rate. Besides the mentioned difference, Cohen's kappa testing confirmed a high qualitative agreement between the two methods. Moreover, the numerical results correlate strongly.

The relevance of the improvement by ChLIA presented here for routine diagnostics is underlined by a previous report about negative ELISA results in anti-GBM disease patient. Epitope accessibility was also cited as a cause of the false-negative findings in a previous study (13). Recently, the same authors evaluated another commercial chemiluminescence assays (CIA on a BIO-FLASH instrument, INOVA, San Diego, USA) in comparison to the Anti-GBM ELISA (IgG) from EUROIMMUN and another inhouse ELISA (19). CIA and the two different ELISA were fully consistent with regard to qualitative results in a cohort of confirmed anti-GBM disease samples. However, correlation between CIA and the Anti-GBM ELISA (IgG) (EUROIMMUN) was lower (*r*_s_ = 0.458) compared to the data presented here (*r*_s_ = 0.962). Moreover, a lower positive agreement (70%) and a higher negative agreement (98.6%) between CIA and ELISA was reported for a cohort of suspected anti-GBM disease patients. Although a comparison with the data presented here is not possible, both reports are consistent with the advantages of chemiluminescence immunoassays.

Given the high sensitivity and specificity of the newly developed Anti-GBM ChLIA (IgG), it provides a valuable tool for supporting anti-GBM disease diagnosis. The fully automated random-access processing makes the Anti-GBM ChLIA (IgG) a very efficient, flexible, and reliable diagnostic immunoassay. Future studies with samples from patients under treatment are necessary to further analyze the assay performance and its relevance in monitoring anti-GBM levels in follow-ups.

## Data Availability Statement

The raw data supporting the conclusions of this article will be made available by the authors, without undue reservation.

## Author Contributions

LH, CD, and WS conceived and designed the study. LH and CD contributed to the generation and collection of data. AK, LH, CD, and WS contributed to analysis and interpretation of data and wrote the paper. All authors contributed to the article and approved the submitted version.

## Funding

This study was funded by EUROIMMUN.

## Conflict of Interest

AK, LH, and CD are employees of EUROIMMUN, a company that develops and manufactures immunoassays for the detection of disease-associated antibodies. WS is a board member of EUROIMMUN.

## Publisher's Note

All claims expressed in this article are solely those of the authors and do not necessarily represent those of their affiliated organizations, or those of the publisher, the editors and the reviewers. Any product that may be evaluated in this article, or claim that may be made by its manufacturer, is not guaranteed or endorsed by the publisher.
